# Gene model for the ortholog of *snu* in *Drosophila funebris*

**DOI:** 10.17912/micropub.biology.002347

**Published:** 2026-09-29

**Authors:** Braiden S. McAlpin, Pablo Chialvo, Clare Scott Chialvo

**Affiliations:** 1 Biology, Appalachian State University, Boone, North Carolina USA

## Abstract

We developed a gene model for the ortholog of the *Drosophila melanogaster* gene *snustorr* ( *snu* ) in the ASM1890182v1 Genome Assembly (GenBank Accession: GCA_018901825.1 ) of *Drosophila funebris* . This ortholog was characterized as part of a developing dataset for a comparative study of detoxification gene family evolution in the *immigrans* - *tripunctata* radiation of the genus *Drosophila* using an adapted Genomics Education Partnership gene annotation protocol for Course-based Undergraduate Research Experiences.

**Figure 1. Genomic neighborhood and gene model for snu ortholog in D. funebris:** (A) **Synteny comparison of the genomic neighborhoods for *snu* in *Drosophila melanogaster* and *Drosophila funebris* .** Thin underlying arrows indicate which DNA strand the target gene, *snu,* is located on in *D. melanogaster* (top) and *D. funebris* (bottom). The thin arrow pointing to the right indicates that *snu* is on the positive strand in *D. melanogaster* , and the thin arrow pointing to the left indicates that *snu* is on the negative strand in *D. funebris* . The wide gene arrows pointing in the same direction as *snu* are on the same strand relative to the thin underlying arrows, while wide gene arrows pointing in the opposite direction of *snu* are on the opposite strand relative to the thin underlying arrows. White gene arrows in *D. funebris* indicate orthology to the corresponding gene in *D. melanogaster* , while black gene arrows indicate non-orthology. Gene symbols given in the *D. funebris* gene arrows indicate the orthologous gene in *D. melanogaster* , while the locus identifiers are specific to *D. funebris* . (B) **Gene Model in GEP UCSC Track Data Hub** (Raney et al., 2014). The coding-regions of *snu* in *D. funebris* are displayed in the User Supplied Track (red); coding sequences (CDS) are depicted by thick rectangles and introns by thin lines with arrows indicating the direction of transcription. Subsequent evidence tracks include Spaln of *D. melanogaster* Proteins (purple, alignment of Ref-Seq proteins from *D. melanogaster* ), Coding Regions Predicted by Augustus (dark blue), GeMoMa (teal), and NSCAN PASA-EST (dark green), and RNA-Seq from mixed sex adult flies (brown; alignment of Illumina RNA-Seq reads from *D. funebris* – Erlenbach et al., 2023). (C) **Dot Plot of snu-PA in *D. melanogaster* ( *x* -axis) vs. the orthologous peptide in *D. funebris* ( *y* -axis).** Amino acid number is indicated along the left and bottom; CDS number is indicated along the top and right, and CDSs are also highlighted with alternating colors. Line breaks in the dot plot indicate areas of with low sequence identity between species.

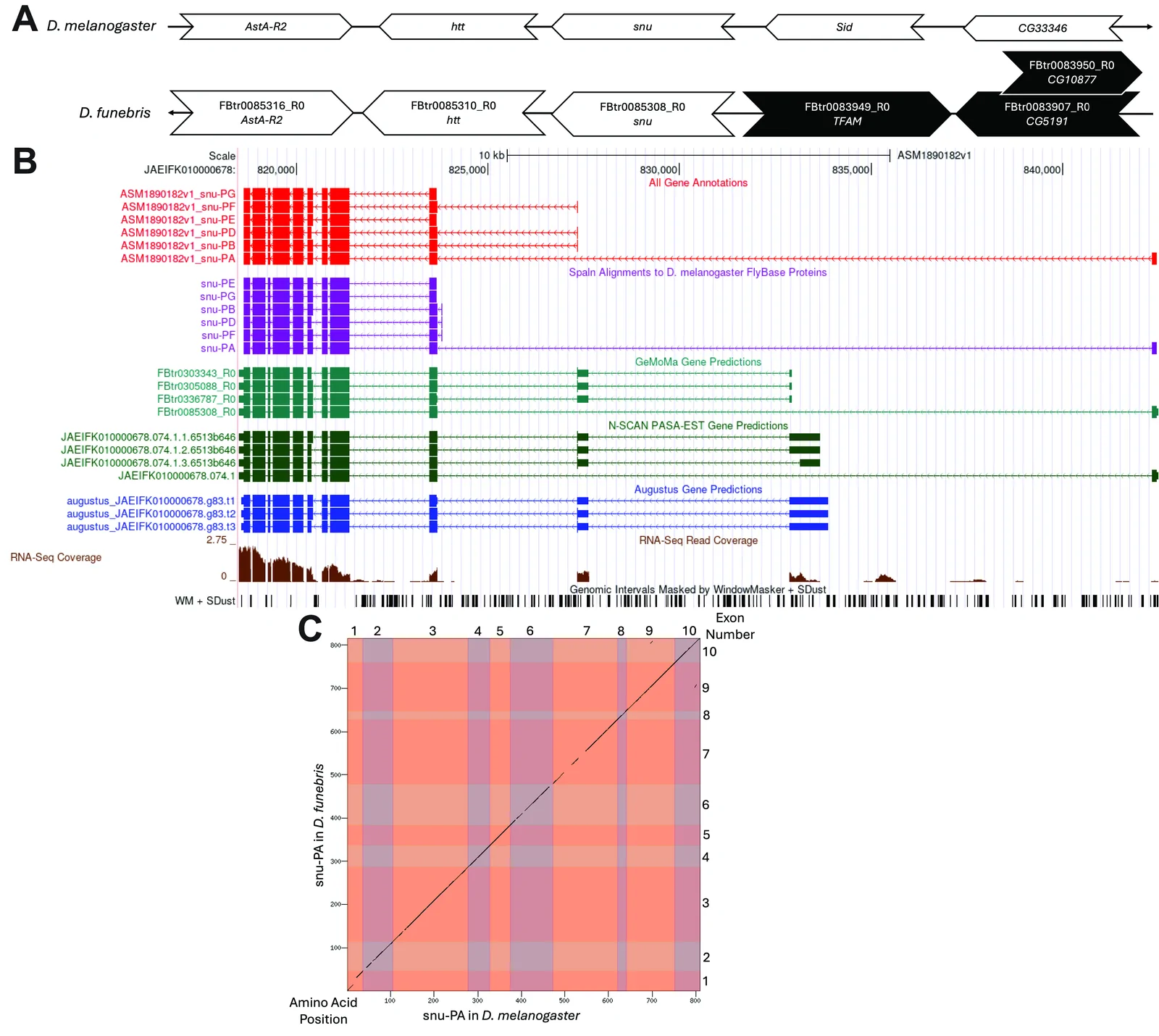



## Description

***Introduction***

**Table:** 

*This article reports a predicted gene model generated by undergraduate work using a structured gene model annotation protocol defined by the Genomics Education Partnership (GEP; thegep.org) for Course-based Undergraduate Research Experience (CURE). The following information in quotes may be repeated in other articles submitted by participants using the same GEP CURE protocol for annotating Drosophila species orthologs of Drosophila melanogaster detoxification genes.* “Within insects, detoxifying xenobiotics and host secondary metabolites is a three-phase process that involves functionalization, conjugation, and excretion of these compounds. Expansions of known detoxification gene families ( *e.g.* , cytochrome P450s) are associated with diet breadth and insecticide resistance (Ranson et al., 2002; Després et al., 2007; Rane et al., 2016). With the increasing availability of high-quality genomes for non-model organisms, including *Drosophila* species beyond *D. melanogaster* , it is now possible to perform large scale comparative studies (Robinson et al., 2011; Kim et al., 2021; Threfall and Baxter, 2021). Careful manual annotation and curation of gene models can improve upon computational gene predictions in non-model species, which aids the accuracy of studies on gene and genome evolution (Mudge and Harrow, 2016; Tello-Ruiz et al., 2019). To aid in these annotations, the Genomics Education Partnership (thegep.org) developed a curriculum involving web-based tools that allow undergraduates to engage in authentic course-based research focused on manually annotating genes in non-model species (Rele et al., 2023). The orthologous gene models, including the one presented here, then provide a reliable basis for further evolutionary genomic analyses when made available to the scientific community. The gene ortholog described here in *D. funebris* for *snustorr* ( *snu* ), a member of the ABC transporter gene family, was characterized as part of a developing dataset for a comparative study of detoxification gene families in the *immigrans* - *tripunctata* radiation of the genus *Drosophila* .” (Williams et al., 2026) “ *Drosophila funebris* (Fabricius, 1787) is a member of the *funebris* species group, which occurs in the *immigrans-tripunctata* radiation of the *Drosophila* subgenus (Bächli, 2005; ICZN, 2010). It is also the type species of the genus *Drosophila* . This species is a globally distributed human commensal (Grimaldi, 2022). While *D. funebris* feeds on shelf fungi and fruit (Kimura et al., 1977; Prigent et al., 2003), it does not tolerate the mushroom toxin α-amanitin (Stump et al., 2011; Erlenbach et al., 2023).” (McDonald et al., 2026). "ATP-binding cassette (ABC) transporters constitute one of the largest protein superfamilies across all domains of life, functioning as primary-active transporters that use the energy of ATP hydrolysis to move a broad range of substrates across lipid membranes (Higgins, 1992; Dean et al., 2001). In the context of xenobiotic metabolism, ABC transporters mediate phase III detoxification by excreting toxins along with phase I and phase II metabolites out of cells. Their overexpression is a recurrent mechanism of insecticide and multidrug resistance in insects (Merzendorfer, 2014; Dermauw and Van Leeuwen, 2014)." (Dieterle et al., 2026) *snustorr* ( *snu* ) is a member of the ABCH ATP-binding cassette transporter subfamily of ABC transporters. It is required for cuticle development and localizes to the apical plasma membrane and cytoplasmic vesicles, consistent with a role in exporting lipids to build the epidermal barrier (Zuber et al., 2018; Wang et al., 2020). Because the cuticular barrier is the insect's first defense against xenobiotic penetration, *snu* is considered part of the "Phase 0" detoxification program (Gao et al., 2022). Transcripts are enriched in the embryonic tracheal system and epidermis (Öztürk-Çolak et al., 2024).

We propose a gene model for the *D. funebris* ortholog of the *D. melanogaster* *snustorr* ( *snu* ) gene. The genomic region of the ortholog corresponds to the GeMoMa FBtr0085308_R0 gene prediction in the ASM1890182v1 Genome Assembly of *D. funebris* ( GCA_018901825.1 – Kim et al, 2021). This model is based on mixed sex, adult RNA-Seq data from *D. funebris* (Erlenbach et al., 2023; https://doi.org/10.5061/dryad.hdr7sqvq2) and *snu* in *D. melanogaster* using FlyBase release FB2024_02 ( GCA_000001215.4 ; Öztürk-Çolak et al., 2024).

***Synteny***

The reference gene, *snu,* occurs on chromosome 3R in *D. melanogaster* and is flanked upstream by *huntingtin* ( *htt* ) and *Allatostatin A receptor 2* ( *AstA-R2* ) and downstream by *Stress induced DNase* ( *Sid* ) and *CG33346* . The *tblastn* search of *D. melanogaster* snu-PA (query) against the *D. funebris* (GenBank Accession: GCA_018901825.1 ) Genome Assembly (subject) placed the putative ortholog of *snu* within contig_683 ( JAEIFK010000678 ) which corresponds to GeMoMa gene prediction FBtr0085308_R0 (E-value: 0; percent identity: 92.53% as determined by *blastp* ). The putative ortholog is flanked downstream by FBtr0083949_R0, FBtr0083907_R0, and FBtr0083950_R0 (nested) which correspond to *mitochondrial transcription factor A* ( *TFAM* ), *CG5191* , and *CG10877* in *D. melanogaster* (E-value: 7e-142, 0, 0; identity: 69.42%, 96.97%, 70.79% respectively, as determined by *blastp* ; Figure 1A ; Altschul et al., 1990). The putative ortholog of *snu* is flanked upstream by FBtr0085310_R0 and FBtr0085316_R0, which correspond to *htt* and *AstA-R2* in *D. melanogaster* (E-value: 0 and 6e-179; identity:66.34% and 70.95%, respectively, as determined by *blastp* ). The putative ortholog assignment for *snu* in *D. funebris* is supported by the following evidence: the *tblastn* results are of good quality, and all coding sequences (CDS) and isoforms found in *D. melanogaster* also appear to be present in *D. funebris.* Though the gene predictions immediately downstream of the putative *snu* ortholog are not conserved, they do appear in the same order and orientation much further downstream in *D. melanogaster* . Conversely, the upstream genetic neighborhood of the putative ortholog is conserved. As such, we conclude that FBtr0085308_R0 is an ortholog of *snu* in *D. funebris* ( Figure 1A ).

***Protein Model***

*snu* in *D. funebris* has ten CDS within the genomic sequence. All six isoforms (PA-PE, Figure 1B ) are translated from unique messenger RNAs. Relative to the ortholog in *D. melanogaster* , the CDS number and protein isoform count are conserved. The sequence of snu-PA in *D. funebris* has 92.5% identity (96.1% similarity) with the protein-coding isoform snu-PA in *D. melanogaster* , as determined by *blastp* ( Figure 1C ). Coordinates of this curated gene model are archived in the CaltechDATA repository (see “Extended Data” section below).

## Methods

The annotation methods used in this project are adapted from those described in Rele et al. (2023), which includes algorithms, database versions, and citations for the complete annotation process developed for the Pathways Project. The methods for the current project are detailed in brief below with notes on significant differences between this protocol and the one described in Rele et al. (2023). The students use the GEP instance of the UCSC Genome Browser v.435 (https://gander.wustl.edu ; Kent et al., 2002; Raney et al., 2024) to examine the genomic neighborhood of their reference detoxification gene in the *D. melanogaster* genome assembly (Aug. 2014; BDGP Release 6 + ISO1 MT/dm6). Students obtain the protein sequence for the *D. melanogaster* target gene for a given isoform and use a *tblastn* search of the sequence against their target *Drosophila* species genome assembly ( *D. funebris* ( GCA_018901825.1 – Kim et al, 2021)) on the NCBI BLAST server (https://blast.ncbi.nlm.nih.gov/Blast.cgi, Altschul et al., 1990) to identify the putative ortholog location. Students compare the genomic neighborhood of the putative ortholog to that of the reference gene in *D. melanogaster* . This local synteny analysis includes a minimum of two upstream and two downstream genes relative to the potential ortholog. As no RefSeq protein data is available for these species, comparisons are based on gene predictions that correlate with gene expression data in the putative ortholog neighborhood. Using the multiple alignment tracks feature in the Genome Browser, students examine other sets of genomic evidence, including Spaln alignment of *D. melanogaster* proteins, multiple gene prediction tracks (e.g., GeMoMa, Augustus, NSCAN PASA-EST), and mixed sex RNA-Seq adult expression data from the target species generated by Erlenbach et al. (2023; https://doi.org/10.5061/dryad.hdr7sqvq2). Information on the genomic structure information (e.g., CDSs, intron-exon number, number of isoforms) for the reference gene in *D. melanogaster* is retrieved using Gene Record Finder (https://gander.wustl.edu/~wilson/dmelgenerecord/index.html; Rele et al *.,* 2023). To determine approximate splice sites within the target gene, a *tblastn* search using the CDSs from the *D. melanogaste* r reference gene against the putative ortholog location (10kb up- and downstream of the target gene prediction). Coordinates of the CDS(s) are refined by examining aligned RNA-Seq data, identifying canonical splice site sequences, and ensuring the maintenance of an open reading frame. Students confirm the biological validity of their target gene model using the FlySeq Gene Model Checker (https://gander2.wustl.edu/~wilson/genechecker-flyseq/), which compares the hypothesized target gene model's structure and translated sequence against the *D. melanogaster* reference gene. At least two independent models for this gene are generated. These models are reconciled by a third independent researcher to produce the final model presented here. Note: comparison of 5' and 3' UTR sequence information is not included in this GEP CURE protocol.

## Data Availability

Description: Zipped archive containing FASTA, PEP, and GFF of snu model in D. funebris. Resource Type: Model. DOI: https://doi.org/10.22002/tqrd0-6z073
